# General commentary: GQIcombi application to subdue glioma via differentiation therapy

**DOI:** 10.3389/fonc.2024.1466102

**Published:** 2024-12-12

**Authors:** BinBin Yuan, Hui Gong

**Affiliations:** ^1^ Department of Public Relations, Affiliated Haimen Hospital of Xinglin College, Nantong University, Nantong, Jiangsu, China; ^2^ Department of Neurosurgery, Affiliated Haimen Hospital of Xinglin College, Nantong University, Nantong, Jiangsu, China

**Keywords:** glioma, differentiation therapy, drug resistance, social work, tumor heterogeneity

## 1 Introduction

Glioblastoma, particularly glioblastoma multiforme, is one of the most aggressive brain tumors, with limited treatment options. Traditional therapies like surgery, radiation, and chemotherapy show high recurrence rates and poor long-term outcomes (1). Although recent advances in gene therapy, cell therapy, and immunotherapy hold promise, they still face significant challenges (2). The article of Kolesnikova et al., “GQIcombi application to subdue glioma via differentiation therapy”, presents an innovative approach using a cocktail of GQIcombi components to reprogram glioma cell fate. This differentiation therapy inhibited glioma cell proliferation and invasion while inducing apoptosis and differentiation in mature neuronal cells. These findings offer new hope for glioma therapy, which has struggled to effectively target tumor cells without damaging healthy brain tissue.

As social workers, we recognize the psychological burden that glioma patients and their families face (3). Promoting understanding of novel therapies can help alleviate some of this anxiety and offer hope, encouraging informed participation in clinical trials. While the study of Kolesnikova et al. shows promise, it is essential to critically assess its limitations and the broader challenges in glioma therapy.

## 2 Subsection 1

From a neurosurgeon’s perspective, while the study offers exciting preliminary data, there are several considerations regarding its applicability:

### 2.1 Preclinical models and tumor heterogeneity

The study primarily relies on *in vitro* and *in vivo* models to validate GQIcombi’s efficacy. While these models are valuable for early-stage research, they cannot fully replicate the complexity and heterogeneity of human gliomas. Gliomas vary greatly in their genetic and epigenetic makeup, which influences their response to treatment (4, 5). The study’s use of cell culture and rat models provides useful insights, but further research with more diverse human glioma samples is necessary to better predict clinical outcomes. Tumor heterogeneity remains a significant challenge in translating preclinical findings into successful clinical treatments (6).

### 2.2 Off-target effects and specificity of GQIcombi

The GQIcombi cocktail shows potential in targeting glioma cells, but the study does not fully address the possible off-target effects. The GQ structure, while effective in promoting differentiation, could interact with non-target cellular components, leading to unintended consequences (7). It is crucial to assess the specificity of GQIcombi for glioma cells and understand how it may affect normal brain cells. Given the sensitivity of neural tissue, future research should investigate potential off-target effects, especially concerning normal neural stem cells and other healthy brain cells that may be inadvertently impacted by the therapy (8).

### 2.3 Resistance to differentiation therapy

The study does not consider the possibility that glioma cells could develop resistance to GQIcombi over time. Glioma cells, like other cancer cells, are capable of developing resistance to various therapies, including differentiation agents (9). While the study shows short-term efficacy, it remains uncertain whether the therapy will prevent recurrence in the long term. The tumor microenvironment (TME), which includes factors like hypoxia, cytokines, and extracellular vesicles, plays a significant role in tumor progression and resistance (10, 11). Ignoring the TME may lead to an overestimation of the therapy’s effectiveness. Future studies should explore the TME’s role in modulating GQIcombi’s effects and investigate potential resistance mechanisms.

### 2.4 Personalized treatment approaches

Gliomas are highly heterogeneous tumors, and patients may respond differently to treatment based on the genetic and epigenetic profiles of their tumors (12). The study uses a limited set of glioma cell lines (e.g., U87, A172, and U251), which may not fully represent the diversity of glioma subtypes seen in clinical practice. Personalized treatment approaches are essential to optimize the use of GQIcombi. Future research should focus on genetic and molecular profiling to identify which glioma subtypes are most likely to benefit from GQIcombi. Additionally, combining GQIcombi with other treatments, such as immunotherapy or targeted therapies, may enhance its efficacy and reduce the risk of resistance (13).

### 2.5 Impact on normal brain cells and stem cell populations

While GQIcombi induces glioma cells to differentiate into mature neurons, the study does not assess the potential impact on normal neural stem cells. Normal neural stem cells are essential for brain homeostasis, and any disruption to these populations could have long-term effects on brain function (14). It is important for future studies to explore whether GQIcombi affects normal stem cell niches, as unintended consequences could impair brain health or regeneration.

## 3 Subsection 2

From the perspective of social workers

### 3.1 Raising awareness and providing psychological support

The introduction of innovative treatments like GQIcombi offers new possibilities for glioma patients. As social workers, it is crucial to raise awareness about such treatments and their potential benefits. Educating patients on how they can participate in clinical trials or access the latest therapies can help reduce the uncertainty that often accompanies a cancer diagnosis. Psychological support is an essential part of care, addressing patients’ emotional and mental health needs. Reducing anxiety and improving quality of life can empower patients, offering them hope and a greater sense of control over their treatment options (15).

### 3.2 Community support networks

Beyond promoting new treatments, social workers play a vital role in helping glioma patients navigate the healthcare system. This includes assisting with access to medical resources, arranging appointments, and ensuring that patients receive the appropriate medications. Community-based support networks are essential for ongoing care, providing patients with the support they need throughout their treatment journey and reducing feelings of isolation (3).

## 4 Discussion

The study of Kolesnikova et al. introduces an exciting new approach to glioma treatment through GQIcombi, which holds promise for targeting glioma cells and inducing differentiation. However, there are important considerations regarding its applicability to clinical practice. The study’s reliance on preclinical models, its limited exploration of off-target effects, and the lack of attention to potential resistance mechanisms and the tumor microenvironment suggest that the results should be interpreted cautiously. Future research, including large-scale clinical trials, personalized treatment strategies, and the optimization of drug delivery systems, will be essential to fully evaluate the therapeutic potential of GQIcombi in glioma patients. As social workers, we are hopeful that ongoing research will lead to safer and more effective treatments, ultimately improving patient outcomes and quality of life.
